# Prothèse totale de hanche dans les séquelles de coxalgie: à propos de 10 cas

**DOI:** 10.11604/pamj.2016.24.105.5149

**Published:** 2016-05-31

**Authors:** Aniss Chagou, Abdelatif Benbouha, Abdelkarim Rhanim, Abdou Lahlou, Mohammed Saleh Berrada, Moradh El yaacoubi

**Affiliations:** 1 Service de Traumatologie-Orthopédie, Centre Hospitalier Universitaire Rabat Université Mohammed V, Rabat, Maroc

**Keywords:** Coxalgie, arthroplastie de la hanche, chimiothérapie antituberculeuse, Coxalgia, hip arthroplasty, anti-tuberculous chemotherapy

## Abstract

La coxalgie entraine des destructions ostéo-cartilagineuses de l'articulation de la hanche, ces lésions sont responsables de douleurs intenses provoquant des gènes fonctionnelles et des limitations dans la vie quotidienne. Leur traitement chirurgical est encore mal codifié. Notre objectif est de montrer à travers cette étude l'intérêt de l'arthroplastie totale de hanche associée à la chimiothérapie antituberculeuse pour l'amélioration de la qualité de vie des patients. Nous rapportons une étude rétrospective portant sur 10 cas de prothèses totales de hanche posées sur séquelles de coxalgie au service de traumatologie-orthopédie du centre hospitalier universitaire Rabat de 2002 à 2011. L’âge moyen de nos patients est de 38 ans. La découverte de la coxalgie s'est faite dans des circonstances différentes en fonction des patients. La voie d'abord que nous avons utilisé est exclusivement postéro-externe de Moore. Les prothèses posées ont toute été cimentées. Quatre patients ont nécessité la reconstruction du cotyle. La biopsie peropératoire est positive chez un patient, négative chez les neuf restants. Tous les patients ont été mis sous traitement antituberculeux. Aucune récidive n'a été notée après un recul minimal de 3 ans. Les résultats selon la cotation de Merle d'Aubigné sont jugés bons. En cas de destruction osseuse avancée avec retentissement fonctionnel très mal supporté chez le sujet d’âge mûr, l'arthroplastie totale demeure le traitement de choix des séquelles de coxalgie sous couverture systématique d'une chimiothérapie antituberculeuse efficace.

## Abstract (en)

Coxalgia causes osteocartilaginous destructions of the hip joint; these lesions are responsible for severe pain leading to functional discomfort and limitations in everyday life. Their surgical treatment is still not well established. The aim of our study was to demonstrate the interest of total hip arthroplasty associated with anti-tuberculous chemotherapy to improve patients’ quality of life. We report a retrospective study of 10 cases of total hip replacement following coxalgia at the Department of Orthopaedics and Trauma of Rabat University Hospital from 2002 to 2011. The average age of our patients was 38 years. The discovery of coxalgia was made under different circumstances according to the patients. The surgical approach we used was exclusively posterolateral (Moore). All prostheses were cemented. Four patients required acetabulum reconstruction. Intraoperative biopsy was positive in a patient, negative in the remaining nine. All patients underwent anti-tuberculous treatment. No recurrence was noted after a minimum follow-up of 3 years. Results according to Merle d'Aubigné score were considered good. In case of advanced bone destruction with badly tolerated functional impact in mature subject, total hip arthroplasty always associated with effective anti-tuberculous chemotherapy is the treatment of choice for sequelae of coxalgia.

## Introduction

Non ou mal traitée, la coxalgie évolue vers la destruction ostéo-cartilagineuse avec ankylose, il persiste à long terme des séquelles articulaires plus ou moins sévères sous forme d'une gêne fonctionnelle très mal tolérée combinant une accentuation de la douleur et une limitation majeure de la mobilité articulaire de la hanche ce qui entrave les gestes courants de la vie quotidienne. L'arthroplastie de la hanche en redonnant l'indolence et la mobilité transforme la vie de ces patients.

## Méthodes

Nous rapportons les résultats d'une étude rétrospective portant sur 10 cas colligés au service d'orthopédie du centre hospitalier universitaire de Rabat entre 2002 et 2011. Le suivi est clinique et radiologique avec un recul de 03 à 08 ans (le recul moyen est de 49 mois). Nos résultats fonctionnels on été jugés selon la cotation de Merle d'aubigné [1].

## Résultats

**Figure 1:**
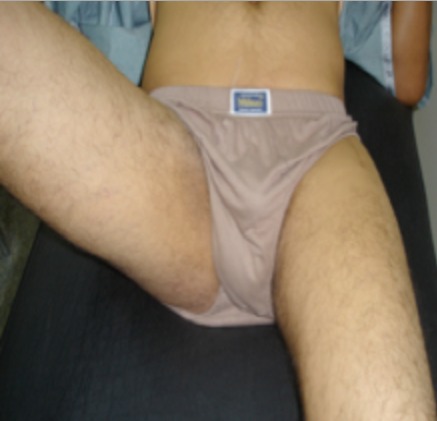
Photo préopératoire, le patient a une bonne abduction à droite, une abduction presque nulle à gauche

**Figure 2:**
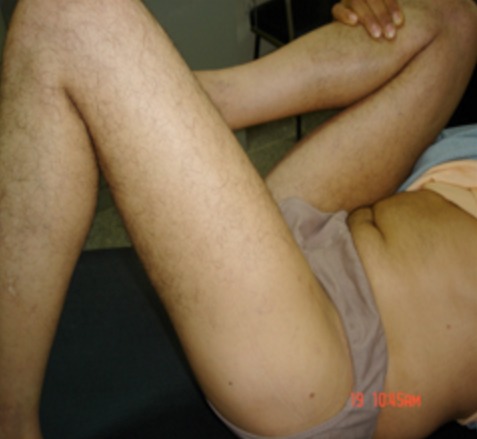
Photo préopératoire, le patient a une flexion complète à droite, à gauche la flexion est limitée à 45

**Figure 3:**
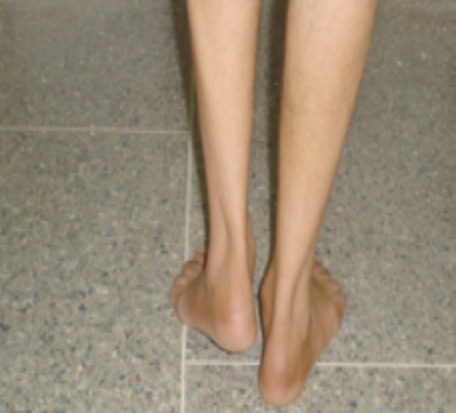
Photo préopératoire, le patient a un raccourcissement du membre inférieur gauche avec inégalité de longueur des deux members

**Figure 4:**
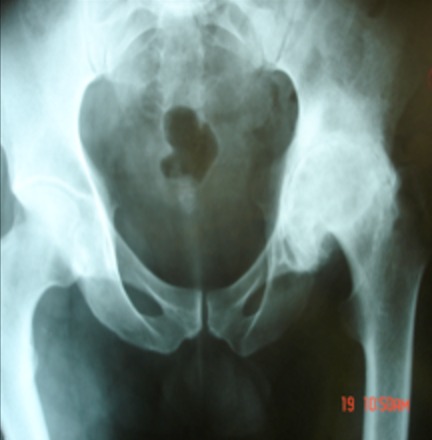
Radiographie préopératoire, montrant la destruction ostéo-cartilagineuse à gauche avec pincement globale de l‘interligne articulaire coxo-fémorale, érosion sous chondrale, déformation en forme de champignon de la tête fémorale avec ascension du grand trochanter

**Figure 5:**
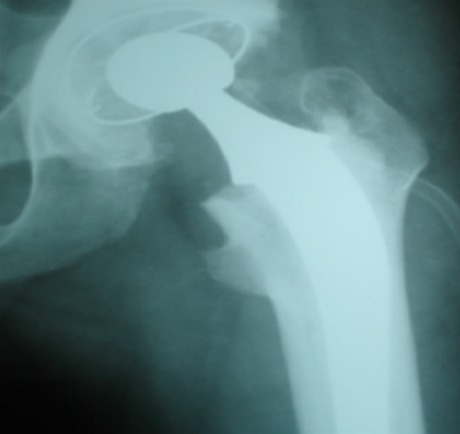
Radiographie de contrôle montrant la prothèse totale de la hanche en place

Nos résultats étaient comme suit: nous avons opéré 4 femmes et 6 hommes d'un âge moyen de 37 ans avec des extrêmes allant 21 et 52 ans. La découverte de la coxalgie s'est faite dans des circonstances différentes en fonction des patients, huit patients ont rapporté une notion de coxalgie traitée dans l'enfance. Alors qu'une patiente a rapporté une notion d'ostéoarthrite de la hanche fistulisée. La découverte de la coxalgie chez la patiente restante s'est faite durant la grossesse, la patiente a été traitée après l'accouchement. Tous les patients inclus dans notre étude présentaient un stade III ET VI de Martini. Nous avons utilisé exclusivement la voie d'abord postéro-externe de Moore et toutes les prothèses posées ont été cimentées. Quatre patients ont nécessité la reconstruction du cotyle par autogreffe osseuse prélevée à partir de la tête fémorale avec butée postéro-supérieure (2 cas) et greffe du fond du cotyle (2 cas). Toutes les prothèses utilisées sont cimentées. La biopsie peropératoire était positive chez un patient, négative chez les 9 autres. Tous les patients ont été mis sous traitement antituberculeux en pré et postopératoire, la durée totale du traitement est de 6 mois. Aucune récidive n'a été notée. Le choix d'une arthroplastie totale montre qu'après 3 ans en moyenne de recul (1-6 ans) les résultats fonctionnels appréciées selon la cotation de Merle d'Aubigné restent favorables dans la majorité des cas avec une moyenne de 16,3, les résultats radiologiques sont aussi satisfaisants à l'exception d'un cas de descellement cotyloïdien aseptique qui a nécessité une reprise chirurgicale ultérieure avec mise en place d'un nouveau cotyle. Les Figure 1, Figure 2, Figure 3 montrent la raideur de la hanche chez un patient en préopératoire alors que la Figure 4 la destruction articulaires sequellaire de la coxalgie. La Figure 5 montre l'aspect radiologique post-opératoire.

## Discussion

L’âge moyen de nos patients est de 38 ans, il est de 46 ans dans la série d'Eskola [2], de 60 ans dans la série de Kim [3] et de 34 ans dans la série d'Hardinge [4]. Concernant les antécédents, nous avons huit patients qui ont été traités pour une coxalgie à l'enfance, une patiente diagnostiquée au cours de la grossesse et traitée après. Eskola [2] rapporte 16 cas ayant des antécédants de coxalgie traité à l’âge de 12 ans et 2 cas traité à l’âge de 20 ans. Johnson [5] rapporte deux cas de coxalgie diagnostiqué et traité à l’âge de l'enfance. Tous les patients inclus dans notre étude présentaient un stade III ET VI de Martini. Tous les auteurs Neogi al [6]; Kim et al [3]; Johnson [5] ont recruté les mêmes stades car selon Babhulkar et Pande [7] les stades I et II évoluent très bien sous traitement médical anti tuberculeux et ce n'est qu'a partir des stades III et IV que l'ankylose retentit sur le vécu des patients atteints de coxalgie. Tous nos patients sont opérés suivant la voie d abord postéro externe de Moore. Les autres auteurs Hardinge [3], Kim [3] et Johnson [5] ont utilisé une approche latérale avec réalisation d'une ostéotomie du grand trochanter en cas de nécessité. Nous avons utilisé exclusivement des prothèses cimentées. Kim et al [3] rapporte que la prévalence de réactivation de l'infection tuberculeuse aux niveaux des hanches traitées par PTH cimentée était comparable à celle non cimentée. En fait, les deux types de prothèses peuvent être utilisés pour le traitement de coxalgie car la thermo réaction induite par le ciment acrilyque est sans rapport avec la réactivation. Le Tableau 1 synthétise les différents types de prothèses utilisées et le fréquence associée de la réactivation du BK.

**Tableau 1:** Comparaison de la fréquence de rechutes dans les séries en fonction de l'utilisation du ciment. Dans la colonne type de la prothèse, on distingue les prothèses cimentées et non cimentées en fonction des séries alors que la colonne rechute dénombre les cas de rechutes dans les séries

Série	Type de prothèse	rechutes
Eskola [2]	Non cimentées	0 cas
Hardinge [4]	cimentées	0 cas
Kim [3]	cimentées	5 cas
Johnson [5]	cimentées	2 cas
Yoon [8]	2 cimentés, 4 non cimentées	0 cas
Notre série	cimentée	0 cas

Le rôle d'un traitement anti-bacillaire adapté est primordial pour obtenir la guérison de l'infection encore plus que pour éviter sa réactivation par réveil de foyers bacillaires éteints ou quiescents comme le montre les travaux de Johnson [5]. Il insiste sur le fait que même après une longue période de quiescence, il persiste toujours un risque de réactivation de l'infection après la chirurgie. C'est pour cela qu'il préconise de traiter les patients par une chimio prophylaxie antituberculeuse en pré et postopératoire chez tous les patients malgré la négativité des biopsies peropératoire. Alors que pour Kim [3], cette chimio prophylaxie n'est pas nécessaire à moins qu'il existe une suspicion ou preuve d'une infection active et a moins que la durée de la période quiescente soit inférieure à 10 ans. Hardinge [4], de sa part n'accompagnait le remplacement prothétique de l'articulation d'aucune chimiothérapie, il n'a rapporté sur une série de 21 cas aucune récidive de l'infection tuberculeuse. Le Tableau 2 synthétise la fréquence des rechutes après l'utilisation ou non de chimiothérapie associée. Devant ces conduites controversées, nous avons adopté celle de Johnson [5]. Nous n'avons noté aucune récidive sur les 10 cas opérés, un patient présentait une biopsie peropératoire positive. Cette chimio prophylaxie s'est faite selon un schéma court de 6 mois: 2RHZ/ 4RH avec à la phase d'attaque (2 premiers mois): association Rifampicine (R) + Isoniazide (H) + Pyrazinamide (Z) et à la phase d'entretien (restants): association Rifampicine (R) +Isoniazide (H). Alors qu'en pratique de nombreux auteurs recommandent une durée minimale de traitement de 12 mois dans la tuberculose ostéoarticulaire [9]. Actuellement, la nouvelle stratégie adoptée au Maroc vise à réduire la durée du traitement antituberculeux et préconise alors un schéma moderne d'une durée de 6 mois 2RHZE/4RH en suivant les recommandations l'OMS basée sur la stratégie appelée directly observed therapy DOT qui repose sur des prises médicamenteuses contrôlées par une tierce personne. Nos résultats fonctionnels ont été comparés avec ceux des autres séries, le Tableau 3 synthétise cette comparaison.

**Tableau 2:** Comparaison de la fréquence de rechutes dans les séries en fonction de l'utilisation de la chimiothérapie. Dans la colonne chimiothérapie, on distingue les séries où la chimiothérapie a été utilisée alors que dans la colonne rechutes on dénombre les cas constatés après un recul moyen qui est indiqué dans la colonne recul

Série	nombre de cas	chimiothérapie		rechute	recul moyen
		Pré-op	Post-op		
Eskola [2]	18	oui	oui	0 cas	42 mois
Hardinge [4]	34	non	non	0 cas	32 mois
Kim [3]	60	oui	oui	5 cas	96 mois
Notre série	10	oui	oui	0 cas	36 mois

**Tableau 3:** Comparaison des résultats des séries selon la cotation de Merle D'Aubigné. Nos résultats restent globalement satisfaisants

	Hardinge [4]		Kim [3]		Notre série	
	Avant	Après	Avant	Après	Avant	Après
Douleur	3,3	5,9	2,75	4,4	2,32	6,34
Fonction	2,9	5,1	2,35	4,3	2,9	5,20
Mobilité	2,1	5,0	1,85	4	3,81	4,76
PMA moyen	8,3	16	6,95	12,7	9,03	16,3

## Conclusion

En cas de destruction osseuse avancée avec retentissement fonctionnel très mal supporté chez le sujet d’âge mûr, l'arthroplastie totale demeure le traitement de choix des séquelles de coxalgie sous couverture systématique d'une chimiothérapie antituberculeuse efficace afin de prévenir les récidives de l'infection tuberculeuse et donc l’échec de l'arthroplastie.

### Etat des connaissances sur le sujet

- Le rôle d'un traitement anti-bacillaire adapté est primordial pour obtenir la guérison de l'infection tuberculeuse encore plus que pour éviter sa réactivation.
- Les deux types de prothèses peuvent être utilisés pour le traitement de coxalgie car la thermo réaction induite par le ciment acrilyque est sans rapport avec la réactivation.

### Contribution de notre étude a la connaissance

- L'intérêt de l'arthroplastie totale de hanche associée à la chimiothérapie antituberculeuse pour l'amélioration de la qualité de vie des patients.
- La couverture par chimiothérapie antituberculeuse efficace est indispensable afin de prévenir les récidives de l'infection tuberculeuse et donc l’échec de l'arthroplastie.
